# Graves' Disease Presenting with Periodic Paralysis to the Emergency Department

**DOI:** 10.1155/2018/9763452

**Published:** 2018-06-07

**Authors:** Nick Si Rui Lan, P. Gerry Fegan

**Affiliations:** Fiona Stanley Hospital, 11 Robin Warren Drive, Murdoch, 6150, Western Australia, Australia

## Abstract

Thyrotoxic periodic paralysis is an infrequent manifestation of hyperthyroidism and an uncommon cause of muscle weakness in western countries. The diagnosis should be considered in the differential when a patient presents with transient and recurrent weakness associated with hypokalaemia. We present a case of a 26-year-old Asian male presenting with sudden onset muscle weakness affecting predominantly his lower limbs on a background of weight loss. Physical examination demonstrated symmetrical proximal muscle weakness with normal sensation and reflexes. Initial biochemical investigations revealed hypokalaemia, hypomagnesaemia, and hyperthyroidism. Intravenous electrolyte replacement was administered in the emergency department. The patient's symptoms resolved during inpatient admission. Subsequent TSH receptor antibody testing and radionuclide thyroid scan confirmed a diagnosis of Graves' disease. The patient was discharged on antithyroid medication with no further episodes of weakness on follow-up. Therefore, thyrotoxic periodic paralysis can be the presenting feature of previously undiagnosed Graves' disease and should be considered in the differential diagnosis in patients presenting with weakness.

## 1. Introduction

Thyrotoxic periodic paralysis (TPP) is a sporadic condition that presents with transient recurrent muscle weakness and severe hypokalaemia (usually <3.0 mmol/L) in patients with thyrotoxicosis of any origin [1, 2]. The muscle weakness can range from mild weakness to life-threatening flaccid paralysis and can have a sudden onset, with symptoms lasting from hours to days [2, 3]. The condition typically affects proximal muscles more than distal muscles and lower limb muscles more than upper limb [1, 3]. It has no effect on sensory or cognitive function and has variable effects on deep tendon reflexes [1, 3]. Although an uncommon cause of muscle weakness in western countries, it should be considered in the differential diagnosis, especially if provoked with exercise or large meals. We present a case of undiagnosed Graves' disease presenting with thyrotoxic periodic paralysis in the emergency department.

## 2. Case Presentation

A 26-year-old male healthy Asian student presented to an emergency department in Australia with sudden onset generalised weakness affecting predominantly his lower limbs after eating dinner. The patient reported difficulty standing and difficulty lifting his arms without any muscle pain or paraesthesia, headache, or back pain. Although he had experienced multiple similar episodes over the past month, these had been less severe and always self-resolved. It was unclear to the patient if these episodes of weakness were associated with food intake or exercise.

On further questioning, the patient reported 15-kilogram weight loss over the past three months and a four-day history of nonbloody diarrhoea, which resolved one week prior to presentation. He had otherwise been well and was playing soccer regularly. He had no relevant family history, was on no regular medications, and denied using illicit drugs.

On examination, the patient appeared mildly diaphoretic but was afebrile. Heart rate was irregular, at 92 beats per minute and blood pressure was 118/60 mmHg. He had a normal respiratory rate at 18 breaths per minute with oxygen saturations of 98% on room air. Neurological examination revealed symmetrical proximal weakness of upper and lower limbs with normal tone, reflexes, and sensation. The decrease in power was more noticeable in the lower limbs compared to the upper limbs. In addition, there was a mildly enlarged painless thyroid gland, with a slight hand tremor, but no signs of thyroid acropachy or thyroid eye disease. Heart sounds were normal with no murmurs, gallops, or rubs, and lung fields were clear to auscultation and percussion. There was no abdominal tenderness to palpation.

Bedside electrocardiogram revealed atrial flutter with a variable ventricular rate. Initial biochemistry showed hypokalaemia with potassium of 2.3 mmol/L (reference range: 3.5–5.2 mmol/L) and hypomagnesaemia with magnesium of 0.59 mmol/L (reference range: 0.70–1.10 mmol/L). Plasma glucose level was 7.1 mmol/L. Complete blood count, urea and creatinine, liver function, phosphate, and creatinine kinase were unremarkable.

Hypokalaemic periodic paralysis was suspected and thyroid function testing was performed. This revealed hyperthyroidism with a TSH of <0.01 mU/L (reference range: 0.40–4.00 mU/L), T4 of 44 pmol/L (reference range: 9–19 pmol/L), and T3 of 25 pmol/L (reference range: 3.0–5.5 pmol/L), suggesting a diagnosis of TPP. TSH receptor antibodies were elevated at >40 U/L (reference range: <1.8 U/L), confirming a diagnosis of Graves' disease. This was further supported by a radionuclide thyroid scan showing diffuse uptake in the thyroid gland. Antithyroperoxidase antibodies, antithyroglobulin antibodies, and urinary electrolytes were not measured in this case.

Initial treatment in the emergency department included 40 mmol of intravenous KCl and 10 mmol of intravenous MgSO_4_. The patient's weakness resolved during inpatient admission. Potassium was 4.7 mmol/L and magnesium was 0.88 mmol/L on repeat testing 6 hours after the initial biochemical tests. Thereafter, potassium remained normal throughout the admission, without rebound hyperkalaemia or the need for further replacement. The patient was discharged home the next day on Carbimazole 20 mg twice a day and Propranolol 20 mg three times a day. No potassium supplementation was prescribed on discharge and he was not started on anticoagulation for the atrial flutter due to his low risk of thromboembolism.

One month after discharge, the patient reported no further episodes of weakness. His T4 and T3 had normalised, although TSH remained at <0.01 mU/L. His potassium was normal at 4.1 mmol/L. Ongoing monitoring of thyroid function was recommended, with a plan for radioiodine ablation or thyroid surgery if antithyroid medical therapy was unsuccessful. However, further follow-up information was not attainable as the patient returned to his home overseas.

## 3. Discussion

TPP occurs predominantly in people of Asian descent with Graves' disease and is more common in younger men aged 20 to 40 years [1, 3, 4]. This is despite hyperthyroidism and Graves' disease presenting more commonly in women [1–4]. In addition, patients may not have any family history of hyperthyroidism [5]. TPP can occur with any aetiology of thyrotoxicosis, such as toxic adenoma, toxic nodular goitre, thyroiditis and factitious thyroiditis, among others [1, 2].

Although contemporary prevalence data is lacking, it is estimated that TPP affects 1.8–1.9% of people with hyperthyroidism in China and Japan [6, 7]. In a western population, TPP is estimated to affect 0.1–0.2% of people with hyperthyroidism [8]. Nonetheless, due to immigration, awareness of TPP becomes crucial for clinicians in western countries. A Taiwanese study recently demonstrated that whilst patients with TPP may present to an emergency department, the symptoms and signs of hyperthyroidism can be subtle [4]. Thus, a strong clinical suspicion is required.

TPP must be distinguished from hypokalaemic periodic paralysis, which is familial, more commonly seen in Caucasians, and is not associated with hyperthyroidism [1, 3, 5]. Other causes of hypokalaemia (Table 1) or proximal muscle weakness must be considered (Table 2).

Muscle weakness in TPP is thought to occur due to the increased stimulation of the sodium-potassium adenosine triphosphatase (Na^+^-K^+^ ATPase) pump in skeletal muscle by thyroid hormone [1–3, 5]. In addition, patients with TPP may have enhanced catecholamine and insulin response, which also increase the Na^+^-K^+^ ATPase pump activity [1–3, 5]. As such, episodes of weakness may occur during recovery after strenuous exercise, or after a carbohydrate-rich meal [1–4]. Furthermore, it is possible that androgens may increase Na^+^-K^+^ ATPase pump activity, thus explaining the higher incidence in men [2, 5].

The activation of Na^+^-K^+^ ATPase leads to a shift of potassium ions intracellularly, resulting in hypokalaemia and hyperpolarisation of muscle cell membranes [1]. As not all patients with hyperthyroidism develop TPP, it is thought that mutations in genes coding for K^+^ efflux channels such as Kir2.6 can be a predisposing factor [1, 5]. The end result is paradoxical depolarisation, inactivation of Na^+^ channels, and muscle inexcitability [1, 5].

The management of TPP includes electrolyte replacement to reverse muscle weakness and treatment of cardiac dysrhythmias, which may be associated with electrolyte imbalance or hyperthyroidism [1–3]. Hypomagnesaemia and hypophosphataemia are commonly concomitant and may also require replacement [1–3]. Although intravenous potassium replacement leads to more rapid resolution of symptoms compared to oral replacement, there is an increased risk of potentially fatal rebound hyperkalaemia, especially at higher doses [1–4]. This is because total body potassium is not reduced, as the transient hypokalaemia seen in TPP is the result of potassium shifting from the extracellular space to the intracellular space [1–4]. As hypokalaemia is not due to potassium losses, urine potassium is typically low [4]. Use of oral potassium supplementation to prevent further episodes of weakness is generally not recommended [1–3].

Propranolol, a nonselective beta-adrenergic blocker, has been shown to be useful in rapidly aborting acute episodes of weakness and preventing further recurrences due to the reduction of beta-adrenergic tone [1–3]. However, it is imperative that the underlying cause for hyperthyroidism is identified and treated promptly, as TPP does not recur once euthyroid [1–4].

## 4. Conclusion

Thyrotoxic periodic paralysis can be the presenting feature of previously undiagnosed Graves' disease and should be considered in the differential diagnosis for patients presenting with weakness.

## Figures and Tables

**Table 1:** Causes of hypokalaemia.

Urinary losses
Diuretics
Hypomagnesaemia
Bartter and Gitelman syndromes
Gastrointestinal losses
Vomiting
Diarrhoea or laxative abuse
Decreased potassium intake
Poor oral intake
Hyperaldosteronism
Primary hyperaldosteronism
Secondary hyperaldosteronism
Others (e.g. excessive liquorice intake)
Shift of potassium into cells
Metabolic alkalosis
Excessive insulin
Hypo/hyperkalaemic periodic paralysis
Thyrotoxic periodic paralysis
Adrenergic stimulation (e.g. beta-agonists or phaeochromocytoma)
Others
Liddle syndrome
Ectopic adrenocorticotrophic hormone

**Table 2:** Causes of proximal muscle weakness.

Myopathy
Endocrinopathy (e.g., thyrotoxic periodic paralysis)
Inflammatory (e.g., myositis)
Infective (e.g., viral hepatitis or human immunodeficiency syndrome)
Genetic (e.g., muscular dystrophy or metabolic storage disorders)
Malignancy-associated
Drug-induced (e.g., statins, corticosteroids or alcohol)
Others
Nervous system disorder
Motor neuropathies
Peripheral nerve disease (e.g., tick paralysis or demyelinating disorders)
Neuromuscular junction disorders (e.g., Myasthenia gravis or botulism)
